# Primary care access to radiology: Characteristics of trauma patients referred to the emergency department

**DOI:** 10.1111/jep.13738

**Published:** 2022-07-18

**Authors:** Renske W. J. Kusters, Nathalie A. L. R. Peters, Frits H. M. van Osch, Petra C. G. Simons, Mark H. H. M. Hulsbosch, Heinrich M. J. Janzing, Dennis G. Barten

**Affiliations:** ^1^ Department of Emergency Medicine VieCuri Medical Center Venlo The Netherlands; ^2^ Department of Clinical Epidemiology VieCuri Medical Center Venlo The Netherlands; ^3^ Department of Epidemiology, Maastricht University NUTRIM School for Nutrition and Translational Research in Metabolism Maastricht The Netherlands; ^4^ Department of Radiology VieCuri Medical Center Venlo The Netherlands; ^5^ Department of Orthopedic Surgery VieCuri Medical Center Venlo The Netherlands; ^6^ Department of Surgery VieCuri Medical Center Venlo The Netherlands

**Keywords:** access to care, Emergency Department, primary health care, radiology, trauma

## Abstract

**Rationale, Aims and Objectives:**

Low‐urgent Emergency Department (ED) attendances are a known contributing factor to ED crowding. In the Netherlands, general practitioners (GPs) have direct access to radiology facilities during office hours. Patients with radiographically confirmed traumatic injuries are subsequently referred to the ED. We analysed these ED trauma patients' characteristics, provided treatments and ED discharge diagnoses to identify the possibility of alternative care pathways.

**Methods:**

Single‐centre retrospective observational study of trauma patients referred to the ED by the radiology department during office hours (January 2017–December 2017). Data were obtained from patient records. Descriptive statistics were used to analyse the extracted data.

**Results:**

A total of 662 patients were included. The median age was 42 years (range: 1–100, interquartile range (IQR): 15–63) and patients presented to the ED with a median delay of 1 day (range: 0–112 days, IQR: 0–5). Most patients were referred for injuries involving the upper extremities (61.5%) and lower extremities (30%). A total of 48 additional diagnoses were made in the ED. The majority of injuries was classified as ‘minor’ (29.5%) or ‘moderate’ (68.3%) on the Abbreviated Injury Scale (AIS). The median length of stay in the ED was 65 min (range: 7–297 min, IQR: 43–102).

**Conclusion:**

Most patients presented with low acuity injuries and often with a notable delay to the ED. This suggests that the majority of these patients do not necessarily need ED treatment, which may provide an opportunity to counter ED crowding.

## 1 INTRODUCTION

Emergency Department (ED) crowding is a growing public health problem. It has been associated with a longer length of stay (LOS) in the ED, treatment delays, overutilization of diagnostic tools, higher hospital admission rates, poor patient outcomes and decreased patient satisfaction.^1^, ^2^, ^3^ Several contributing factors have been identified, including increasing patient complexity, an ageing population, reduced hospital admission capacity and high rates of nonurgent visits and self‐referring patients.^1^ Improving patient flows and redirecting patients with nonurgent complaints to alternative health‐care settings may help to reduce the workload of EDs.

In the Netherlands, the primary care system is well‐developed and accessible 24 h a day. Musculoskeletal trauma is one of the main reasons to visit a general practitioner (GP).^4^ During office hours, every GP has access to radiology services. In case of radiographic abnormalities detected by a radiologist, the patient is subsequently referred to the ED. The GP receives a radiology report with a notice of the ED referral. If an abnormality is ruled out, the GP remains responsible for evaluation and treatment.^5^ In recent years, various GP cooperatives have also gained access to the hospital's radiology facilities, and several studies have examined the impact of this out‐of‐hours access. In line with expectations, those studies showed a decrease of minor traumatic injuries in the ED, suggesting a positive effect on ED crowding.^5^, ^6^ It seems likely that this also applies within office hours, but it has not been assessed yet. Furthermore, little is known on the delay between injury and diagnosis, and on the type of injuries these patients present with. Such information is essential to further improve the efficiency of this current health‐care trajectory.

The aim of this study was to analyse patient characteristics, provided treatments and discharge diagnoses of ED trauma patients referred by the radiology department during office hours. Furthermore, we collected information on additional diagnostics ordered in the ED with resultant extra diagnoses and treatments.

## 2 METHODS

We performed a retrospective observational study of patients presenting to the ED of VieCuri Medical Center, a 500‐bed teaching hospital with a Level 2 trauma centre in the southeast region of the Netherlands. The hospital serves a population of around 280,000 people and has an annual ED census of 25,000 patients. The ED is colocated with a GP cooperative for out‐of‐hours primary care. We included trauma patients that were referred to the ED by the radiology department within office hours from January 1, 2017 through December 31, 2017. To avoid seasonal influences, patient samples comprised all consecutive patients in the first 6 weeks of each quarter of the year. Patients who were referred by the radiology department for nontraumatic reasons and patients who previously expressed objection to study research participation were excluded.

Data were extracted from digital patient charts in the hospital registration system Healthcare information eXchange (HiX, Chipsoft BV) based on ED registration coded with patient origin ʻradiology referral’. The institutional review board of VieCuri Medical Center approved a waiver of consent in view of the retrospective and observational nature of the study. Data were collected anonymously in an encrypted database (Castor Electronic Data Capture [EDC], 2019) and included age, sex, day and time of presentation, delay to presentation (i.e., time of injury until ED presentation), referral indication, waiting and treatment time, total LOS, injury location, affected body part, International Classification of Diseases (ICD) diagnosis, provided treatment and follow‐up location. Injury severity was determined based on triage category according to the Manchester Triage System,^7^ Abbreviated Injury Scale (AIS) (2008 updated version)^8^ and calculated Injury Severity Score (ISS).^9^ AIS classified injuries within one of six ISS body regions (head/neck, face, chest including thoracic spine injuries, abdomen including lumbar spine injuries, extremities including pelvis, external) on a 6‐point scale (1: minor, 2: moderate, 3: serious, 4: severe, 5: critical, 6: maximum).^8^ ISS is calculated by squaring the highest AIS in each of the three most severely injured body regions.^9^ Additional diagnostics ordered in the ED with resultant extra diagnoses were analysed, except for postreduction radiographs. The extent of the radiology order issued by the GP was assessed and was considered complete when it comprised the following information: time of injury, trauma mechanism, specific area of complaint and specific diagnostic question. Descriptive statistics (e.g., medians and interquartile ranges [IQR]) were used to describe patient characteristics, LOS and injury severity. For all data analyses IBM SPSS Statistics Version 26 was used.

## 3 RESULTS

### 3.1 Patient characteristics

A total of 662 patients were included for analysis. Baseline characteristics are shown in Table 1. The median age was 42 years (range: 1–100, IQR: 15–63), with an equal distribution of sex (50.9% male, 49.1% female). Patients presented to the ED with a median delay of 1 day (range: 0–112 days, IQR: 0–5). A total of 19 patients (2.8%) presented to the ED 1 month or more after suffering the injury, mostly because of unexplained persistent pain. Most patients were injured during sports (24.6%) or at home (22.1%). Patients were most likely to present to the ED on Mondays (27.0%) with injuries to the extremities (95.2%). Delays to presentation were similar for all business days. The most common reason for ED referral by the radiology department was the presence of one or more fractures (93.4%). Other referral indications were joint dislocations, the presence of soft tissue injury, suspicion of neurovascular/tendinous injuries, a request for clinical assessment or a combination of indications. The majority of patients (78.5%) was triaged to the lowest triage categories according to the Manchester Triage System (blue, i.e., ‘nonurgent’ or green, i.e., ‘standard’). The radiology order issued by the GP was considered incomplete in 73% of cases. Of the essential order elements, the timing of injury was most often missing (63%), followed by specific area of complaint (37%) and trauma mechanism (30%).

**Table 1:** Baseline characteristics

	% (*N*)
*Gender*	
Male	50.9 (337)
Female	49.1 (325)
*Age (years)*	
0–18	32.0 (212)
19–69	49.8 (330)
70–100	18.1 (120)
*Injury location*	
Sports/hobby	24.6 (163)
Home	22.1 (146)
Traffic	17.5 (116)
School	5.7 (38)
Work	6.0 (40)
Other	24.0 (159)
*Affected body part*	
Upper extremities	61.5 (407)
Lower extremities	33.7 (223)
Chest	2.4 (16)
Abdomen	1.4 (9)
Face	0.6 (4)
Head/neck	0.5 (3)
External	0 (0)
*Referral indication*	
Fracture(s)	93.4 (618)
Request for clinical assessment	2.0 (13)
Combination of indications	1.7 (11)
Other	1.2 (8)
Neurovascular/tendinous injury	0.9 (6)
Joint dislocation	0.8 (5)
Soft tissue injury	0.2 (1)
*Day of ED presentation*	
Monday	27.0 (179)
Tuesday	18.4 (122)
Wednesday	17.5 (116)
Thursday	16.9 (112)
Friday	18.7 (124)
Saturday	1.4 (9)
*Triage category*	
Green (‘standard’)	69.0 (457)
Blue (‘nonurgent’)	9.5 (63)
Yellow (‘urgent’)	11.0 (73)
Orange (‘very urgent’)	0.5 (3)
Red (‘immediate’)	0.0 (0)
Not specified	10.0 (66)

### 3.2 Injury characteristics

**Figure 1:**
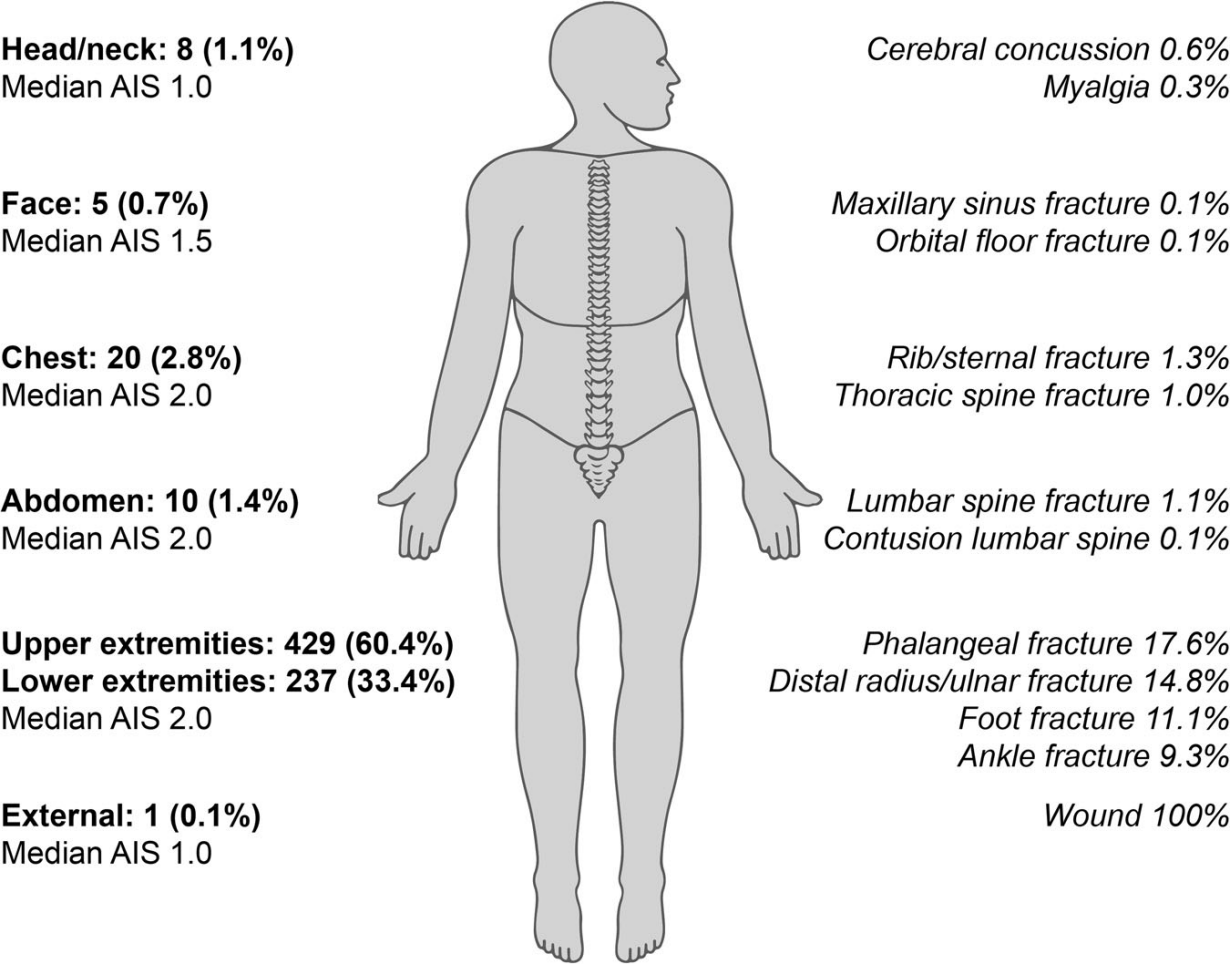
Injury characteristics (multiple diagnoses possible per patient; a total of 710 ED diagnoses in 662 patients). Left: number of diagnoses within ISS body regions (with % of total diagnoses) and median AIS. Right: most common diagnoses (in % of total diagnoses) within accompanying ISS body region on the left. AIS, Abbreviated Injury Score; ISS, Injury Severity Score.

In total, 710 ED diagnoses were made in 662 patients. Most patients were referred for injuries involving the upper extremities (407 of 662 patients; 61.5%). Phalangeal fractures (17.6% of 710 diagnoses), distal radius and/or ulnar fractures (14.8% of all diagnoses) and hand fractures (10.4% of all diagnoses) were most common. Thirty‐four per cent of referrals (223 of 662 patients) concerned injuries in the lower extremities; most frequently foot fractures (11.1% of 710 total diagnoses), ankle fractures (9.3% of all diagnoses) and foot phalangeal fractures (4.9% of all diagnoses). The majority of injuries was classified as ‘minor’ (AIS score 1/6) and ‘moderate’ (AIS 2/6) in all anatomical regions, 29.5% and 68.3%, respectively. A total of 15 chest injuries and injuries involving the extremities were classified as ‘serious’ (AIS 3/6), comprising 2.1% of all injuries. Serious injuries included rib fractures, pneumothorax and proximal femur fractures. No injuries were classified as ‘severe’, ‘critical’ or ‘maximum’ (AIS score 4, 5 and 6, respectively). The median Injury Severity Score (ISS) was 4 (range: 1–10, IQR: 1–4). Overall injury characteristics are shown in Figure 1. A detailed list of diagnoses is included in Supporting Information: File 1.

### 3.3 ED work‐up

**Figure 2:**
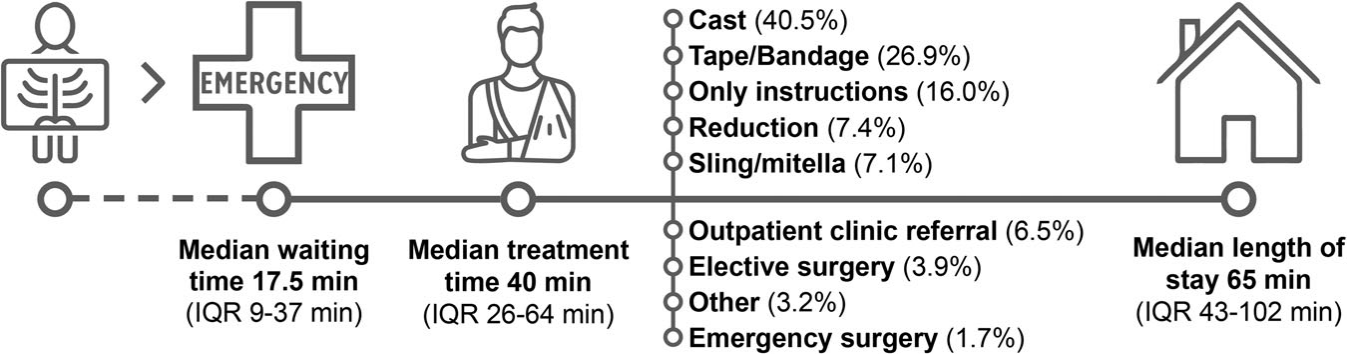
ED timeline. From left to right: patients are referred to the ED from the radiology department, where they are treated for their injuries (listed in % of patients who received this specific treatment) with a subsequent discharge home or hospital admittance. Median ED waiting time, treatment time and LOS are depicted below the timeline. ED, Emergency Department; IQR, interquartile range.

Median LOS was 65 min (range: 7–297 min, IQR: 43–102), median waiting time was 17.5 min (range: 0–168 min, IQR: 9–37) and median treatment time was 40 min (range: 5–261 min, IQR: 26–64). The majority of patients was treated with a cast (40.5%) or tape/bandage (26.9%), and 16% received instructions only. Other ED treatments were sling, wound treatment and joint reductions. One patient (0.2%) underwent procedural sedation because of a shoulder reduction, 11 patients (1.7%) were admitted for (semi)urgent surgery and 43 patients (6.5%) were referred to the traumatology outpatient clinic from the ED because of treatment options that were not available in the ED (such as walking (cast) boot, waterproof cast, orthoses) or because of the delay to presentation. The ED timeline is shown in Figure 2.

**Figure 3:**
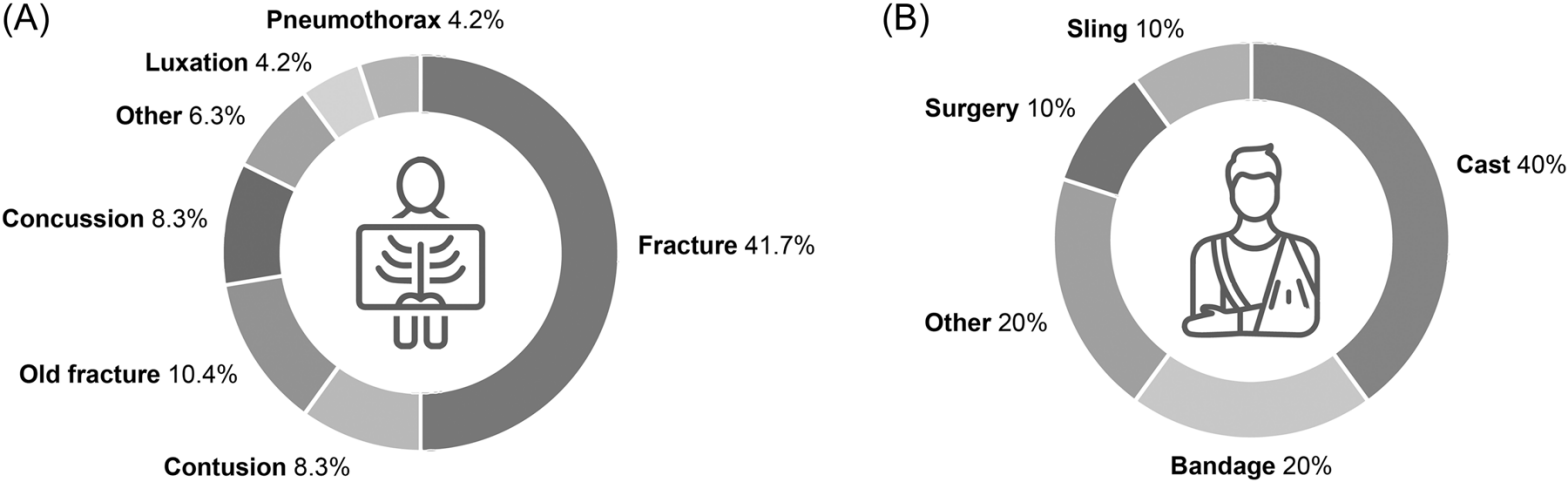
ED work‐up in patients with additional diagnoses. Forty‐eight extra diagnoses were made in the ED (A), leading to 10 additional treatments (B). ED, Emergency Department.

A total of 111/662 patients (16.7%) received additional diagnostics in the ED. Of these, 71 cases were post‐reduction radiographs, which were subsequently excluded from analyses. Additional diagnostics were ordered in the remaining 40 patients (6% of all patients) because of suspected extra injuries or in addition to the original diagnostics. Twenty‐four of these patients (60%, 3.6% of all patients) had an additional radiograph, 14 patients received a computed tomography scan (35%, 2.1% of all patients) and 2 patients received an additional ultrasound during ED evaluation (5%, 0.3% of all patients). Along with the expected diagnoses based on referral indication, 48 new diagnoses were made in the ED (6.8% of all diagnoses), of which 31 were the result of the extra diagnostic tools ordered (4.4% of all diagnoses). Based on AIS scores, extra diagnoses mainly were classified as ‘minor’ and ‘moderate’ (contusions, distal extremity fractures and cerebral concussion), some were classified as ‘serious’ (pneumothorax and pleural effusion). In most patients, these extra diagnoses did not lead to a different treatment because of the minority of the problem, the delay to presentation or because the necessary treatment was already provided for the referral diagnosis. In 10/48 cases (20.8%, 1.4% of all diagnoses) an extra treatment was needed, varying from bandage to a semiurgent surgery. The ED work‐up in patients receiving additional diagnoses and their subsequent extra treatments is shown in Figure 3.

### 3.4 Follow‐up

A total of 532 patients (80.4%) had a follow‐up appointment in the traumatology outpatient clinic. Fourteen patients (2.1%) were admitted in the hospital and 25 patients (3.8%) underwent semielective surgery within 1 or 2 weeks. Eighty‐one patients (12.2%) did not have follow‐up and 10 patients (1.5%) were referred back to primary care for follow‐up.

## 4 DISCUSSION

In this study, patients referred to the ED with radiographically proven traumatic injuries following GP's access to radiology were analysed. Injuries generally were of low acuity and patients presented with varying but considerable delays. Patients often received treatments of low complexity or instructions only and follow‐up was not always indicated. This suggests that the majority of these patients do not necessarily need ED treatment, which may provide an opportunity to counter ED crowding and may help to provide the right care in the right place at the right time.

Although the causes of ED crowding are multifactorial and can be related to increased input (for example, high influx of patients), reduced throughput (for instance, bad logistics) and reduced output (for instance, reduced hospital bed capacity), several reports have shown that patients with nonurgent ED attendances are a contributing factor.^1^, ^2^, ^10^, ^11^, ^12^ Furthermore, poor access to primary care is a critical driver of high ED volumes.^1^, ^10^ In the Netherlands, many EDs are colocated with GP cooperatives, forming so‐called emergency care access points (ECAPs) after hours. Thijssen et al.^13^, ^14^, ^15^ observed that these ECAPs were associated with a decrease in self‐referred ED patients and an increase in hospital admission rates, suggesting an increased efficiency of ED utilization following ECAP implementation. An additional advantage of co‐location is the possibility of direct access to hospital diagnostics, such as radiology facilities. Previous Dutch studies assessed the effect of GPs' out‐of‐hours access to radiology facilities and found a substantial decrease in ED trauma patient referrals and increased patient satisfaction levels.^5^, ^6^, ^16^ Consistent with those studies, the use of diagnostics by GPs in our study seemed adequate. Although 6.8% of patients received an extra ED diagnosis in addition to the radiology department referral indication, they were mainly classified as minor or moderate, and generally had no clinical consequences because of the delay to presentation or because no additional treatment was indicated.

It is well known that ED crowding is associated with delayed treatments, poor patient outcomes, decreased patient satisfaction, higher costs and a high burden for ED staff.^1^, ^2^, ^3^, ^10^ Although the frequency and degree of crowding was not measured in our study and patients already visited the radiology department for diagnostics before their ED visit, the median LOS of 65 min is relatively short compared to both nationally and internationally published LOS' in similar sized EDs.^15^, ^17^, ^18^ However, due to an ageing population, staff shortages and other societal developments, Dutch EDs suffer from rising pressures,^18^ which calls for a further improvement of their efficiency. Various strategies to improve patient flows and throughput times in the ED have been evaluated for years,^1^, ^2^ particularly for nonurgent patients. For example, fast‐track units have been introduced in the ED, where patients with low‐acuity medical conditions are assessed, treated and discharged without diverting resources from patients with high‐acuity conditions.^19^, ^20^ Despite the reported positive effect of fast‐tracks on waiting times and LOS for low‐acuity patients,^20^ high volumes of nonurgent patients still lead to an additional risk of crowding. Moreover, Wickman et al.^21^ found that ED crowding leads to an increased LOS for the overall ED population, both low‐ and high‐acuity. Gill et al.^19^ investigated fast‐track patients with a LOS greater than 4 h and identified several important contributing factors, including radiology turnaround times, month of presentation, type of complaint and type of imaging ordered. These external factors may be difficult to engage. Redirecting nonurgent trauma patients to outpatient or primary care services may therefore be an important tool to reduce pressures on EDs. This was illustrated during the COVID‐19 pandemic, in which a considerable number of Dutch EDs redirected patients with minor traumatic injuries to outpatient departments.^22^ Moreover, in a previous Dutch cohort study it was shown that the direct discharge of simple, stable musculoskeletal injuries from the ED is feasible.^23^

Our findings support the idea that the low‐acuity nature of the injuries and mean delay to presentation for this specific category of trauma patients generally allows for treatment in other health care settings than the ED. An unanticipated finding of our study was the frequent lack of information about time and localization of injury in the radiology order issued by the GP. If radiology orders contain more information, this will possibly help radiologists to even better organize the disposition of trauma patients. If put into practice, strict guidelines on the injuries that can be treated in outpatient departments or by the GP and which patients need to be referred to the ED are warranted. Patients with non‐displaced, closed fractures of the distal extremities might not need ED treatment. Moreover, considering delay to presentation can be useful in determining if referral to alternative health‐care settings is feasible and safe. Even when specific injuries can be treated in alternative health‐care settings, there might be a benefit of ED evaluation. Based on the preliminary results of this study and accelerated by the impact of the COVID‐19 patient surge, patient routing was adapted in our hospital in March 2020. Following strict instructions, numerous patients with radiographically proven traumatic injuries were referred to outpatient services for treatment. An important lesson learned in the expansion of outpatient management was that outpatient clinics also know peak hours at the end of the day, which could be taken into account by scheduling appointment slots for urgent patients. Further research is needed to evaluate this alternate clinical pathway and its effect on patient care, missed injury rates and ED crowding. Future studies should also assess which diagnoses not necessarily need hospital follow‐up, but can be referred back to primary care.

### 4.1 Strengths and weaknesses

A key limitation of this study is the study design, being a single‐centre observational study. We only studied the patients that were referred to the ED. Therefore, the occurrence of selection bias cannot be excluded in case radiographic abnormalities were either missed or regarded ‘minor’ by the radiologist with a subsequent referral back to the GP instead of the ED. Our study population only included patients who presented in the first 6 weeks of each quarter of the year, leading to an underestimation of patients in the ED with minor traumatic injuries on a yearly basis. It can therefore be assumed that adapting patient routing has a greater impact on relieving ED pressures than the study results imply. Furthermore, primary care access to radiology is not available in many countries. The results of this study may therefore have limited generalizability. Nonetheless, ED crowding is universal and this study provides novel insights into the flow of nonurgent trauma patients in the ED. It may help to improve existing care pathways in other emergency health care settings. Furthermore, it supports the common practice of redirecting nonurgent trauma patients to outpatient settings during the first wave of the COVID‐19 pandemic.

## 5 CONCLUSION

Primary care access to radiology aims to only refer patients with radiologically confirmed traumatic injuries to the ED and thereby reduce the number of trauma patient ED referrals. The patients in this cohort presented with a notable delay and injuries were mostly classified as low acuity. This suggests that not all of these patients necessarily need ED treatment, which may provide an opportunity to counter ED crowding by using alternative care pathways, such as outpatient clinics. The safety and feasibility of these pathways should be evaluated in future studies.

## AUTHOR CONTRIBUTIONS

**Renske W.J. Kusters**: conceptualization, data collection, analysis and interpretation of results, visualization, writing draft manuscript; **Nathalie A.L.R. Peters**: review and editing; **Frits H.M. van Osch**: analysis and interpretation of results, review and editing; **Petra C.G. Simons**: review and editing; **Mark H.H.M. Hulsbosch**: review and editing; **Heinrich M.J. Janzing**: review and editing; **Dennis G. Barten**: conceptualization, analysis and interpretation of results, review and editing, supervision.

## CONFLICT OF INTEREST

The authors declare no conflict of interest.

## Supporting information

Supporting information.

## Data Availability

The data that support the findings of this study are available from the corresponding author upon reasonable request.
