# Supplementary material for: Primary care access to radiology: Characteristics of trauma patients referred to the emergency department
Source: J Eval Clin Pract. 2022 Jul 18;29(1):101–7. doi: 10.1111/jep.13738 (PMC10084186; doi:10.1111/jep.13738)
Supplement: Supplementary file 1 — Supporting information. [file JEP-29-101-s001.docx]

**Supplementary information file 1: List of all diagnoses**

| **ISS body region** | **Diagnosis** | **Primary** | **Extra ED** | **Total** |
| --- | --- | --- | --- | --- |
|  |  | **diagnosis (N)** | **diagnosis (N)** | **N (%)** |
| **Head/neck** | **Cerebral concussion** | **0** | **4** | **4 (0.6)** |
|  | Myalgia (neck) | 2 | 0 | 2 (0.3) |
|  | Old cervical spine fracture | 1 | 0 | 1 (0.1) |
|  | Dislocation facet joint cervical spine | 0 | 1 | 1 (0.1) |
| **Face** | **Maxillary sinus fracture** | **1** | **0** | **1 (0.1)** |
|  | **Orbital floor fracture** | **0** | **1** | **1 (0.1)** |
|  | Mandible fracture | 1 | 0 | 1 (0.1) |
|  | Zygoma fracture | 1 | 0 | 1 (0.1) |
|  | Nasal fracture | 1 | 0 | 1 (0.1) |
| **Chest** | **Rib/sternal fracture(s)** | **9** | **0** | **9 (1.3)** |
|  | **Thoracic spine fracture** | **7** | **0** | **7 (1.0)** |
|  | Pneumothorax | 0 | 2 | 2 (0.3) |
|  | Pleural effusion | 0 | 1 | 1 (0.1) |
|  | Contusion thoracic spine | 0 | 1 | 1 (0.1) |
| **Abdomen** | **Lumbar spine fracture** | **8** | **0** | **8 (1.1)** |
|  | Old lumbar spine fracture | 0 | 1 | 1 (0.1) |
|  | Contusion lumbar spine | 1 | 0 | 1 (0.1) |
| **Upper extremities** | **Finger phalangeal fracture** | **120** | **5** | **125 (17.6)** |
|  | **Distal radius and/or ulnar fracture** | **104** | **1** | **105 (14.8)** |
|  | **Hand fracture** | **69** | **5** | **74 (10.4)** |
|  | Elbow fracture | 50 | 3 | 53 (7.5) |
|  | Clavicle fracture | 24 | 0 | 24 (3.4) |
|  | Shoulder fracture | 18 | 1 | 19 (2.7) |
|  | Contusion/distorsion | 10 | 6 | 16 (2.3) |
|  | Old fracture | 3 | 1 | 4 (0.6) |
|  | Glenohumeral joint luxation | 3 | 0 | 3 (0.4) |
|  | Scapula fracture | 1 | 0 | 1 (0.1) |
|  | AC luxation | 1 | 0 | 1 (0.1) |
|  | MCP subluxation | 1 | 0 | 1 (0.1) |
|  | Tendinogenous mallet finger | 1 | 0 | 1 (0.1) |
|  | Muscle/tendon tear | 2 | 0 | 2 (0.3) |
| **Lower extremities** | **Foot fracture** | **77** | **2** | **79 (11.1)** |
|  | **Ankle fracture** | **65** | **1** | **66 (9.3)** |
|  | **Foot phalangeal fracture** | **34** | **1** | **35 (4.9)** |
|  | Contusion/distorsion | 7 | 5 | 12 (1.7) |
|  | Hip fracture | 9 | 0 | 9 (1.3) |
|  | Pelvic ring fracture | 8 | 0 | 8 (1.1) |
|  | Knee fracture | 8 | 0 | 8 (1.1) |
|  | Old fracture | 4 | 3 | 7 (1.0) |
|  | Tibial shaft fracture (crural) | 5 | 0 | 5 (0.7) |
|  | Muscle/tendon tear | 3 | 1 | 4 (0.6) |
|  | Lisfranc luxation | 1 | 1 | 2 (0.3) |
|  | Achilles tendon rupture | 1 | 0 | 1 (0.1) |
|  | Calcaneale apofysitis | 1 | 0 | 1 (0.1) |
| **External** | **Wound** | **0** | **1** | **1 (0.1)** |
|  | Total | 662 | 48 | 710 |
